# Digital Formats for Community Participation in Health Promotion and Prevention Activities: A Scoping Review

**DOI:** 10.3389/fpubh.2021.713159

**Published:** 2021-11-16

**Authors:** Claudia Schroeer, Stephan Voss, Caroline Jung-Sievers, Michaela Coenen

**Affiliations:** ^1^Chair of Public Health and Health Services Research, Institute for Medical Information Processing, Biometry, and Epidemiology – IBE, LMU Munich, Munich, Germany; ^2^Pettenkofer School of Public Health, Munich, Germany

**Keywords:** health promotion, digitalization, public health, participation, community, empowerment

## Abstract

**Objectives:** Digital technologies in public health are primarily used in medical settings and mostly on an individual and passive way of use. There are research gaps on digital media facilitating participation, empowerment, community engagement, and participatory research in community settings. This scoping review aims to map existing literature on digital formats that enable participation in the field of health promotion and prevention in community settings.

**Design:** The databases Medline, EMBASE, and PsycINFO were used to identify studies published from 2010 up to date (date of literature search) onward that used digital formats in all or in the main sequences of the process to enable high levels of participation in health promotion and prevention activities in community settings.

**Results:** This review identified nine out of 11 included studies relevant to the research question. We found five studies that applied qualitative participatory research, two studies on peer support and one study each on empowerment and crowdsourcing. The digital technologies used varied widely and included social media platforms, bulletin boards, online forum webpages, and customized web providers and programs. Most studies mentioned anonymity, flexibility, and convenience as benefits of digital interventions. Some papers reported limitations such as difficulties by interpreting written-only data or the possibility of selection bias due to the digital divide.

**Conclusion:** This scoping review identified only few studies relevant to our objective, indicating an existing gap in research on this topic. Digital formats were found to be particularly suitable for purposes where anonymity and flexibility are beneficial, such as for online peer exchange and peer support programs.

## Introduction

The Ottawa Charta and the Alma Ata Declaration defined community participation as a basic element and key principle for health-promoting activities and programs (1, 2). The Ottawa Charta for Health Promotion was published at the conclusion of the first International Conference on Health Promotion by the World Health Organization (WHO) in Ottawa on November 21, 1986. It is considered one of the most important follow-up documents to the Declaration of Alma-Ata (1978), in which WHO declared basic health care and health promotion to be a fundamental human right. Since these declarations, community participation has become an increasing subject of interest in research and practice (3). Involving members of a community in health promotion may increase the effectiveness of public health interventions and reduce health inequalities for socially disadvantaged groups (4). Participation processes can strengthen social networks that reinforce healthy behaviors and reduce feelings of isolation (5). On an individual level, participation in decision-making processes increase the experience of self-efficacy. Furthermore, participation may initiate a process of empowerment, which enables communities to shape their environment, health, and lives according to their own interests (6). Community-based participatory strategies are based on the assumption that community members are experts of their own lives and thus know best how to improve the health of their community (6).

Communities can be defined as geographic entities ranging from neighborhoods to towns or cities, as well as larger geographic areas (7). Irrespective of geographic boundaries, a community can also be defined by common identity “based on race, gender, religious belief, sexual orientation, or a community-based organization united for a particular cause” (8). Participation was first categorized in Arnstein's ladder of citizen participation. It consists of eight rungs describing a range from non-participation to tokenism to the degree of citizen power (9). This idea has been further developed and adapted in the stage model of participation by Wright et al. (10). They define true participation as involving community members in the decision-making process, including the level in which community members are encouraged to make decisions, have partial decision-making authority, or have decision-making power (11). Below these stages, there are preliminary stages of participation, such as consultation like surveys, or stages that are not considered as participation, such as instruction. Beyond the level of participation goes empowerment by teaching participants necessary skills to initiate and carry out measures in a self-organized manner (12).

Advancing digitalization offers opportunities to exploit new possibilities in the implementation of health promotion and prevention programs (13). Many digital technologies, specifically devices such as computers, mobile phones, and tablets, are already accessible to a wide range of people of all ages and continue to evolve. A review by Clar et al. (14) on existing systematic reviews about the use of digital methods in public health found a wide variety of examples for digital approaches in the public health sector such as eHealth services, social marketing campaigns, apps, video games, telephone interventions, online photovoice, online discussion forums, virtual communities, or online collaborative writing applications. The review found that digital technologies were primarily used in medical settings and mostly in an individual and passive manner. Identified research gaps were digital media facilitating participation, empowerment, community engagement, and participatory research in community settings.

To our best knowledge, there is no review identifying existing studies on digital formats for community participation, irrespective of a specific group or a specific type of community participation. For this reason, we conducted a scoping review to systematically map the research in this area on a broader level and identify core concepts.

This scoping review aims to map existing literature on digital formats that enable participation in the field of health promotion and prevention in community settings to provide a better and more comprehensive understanding of the research area. This review will focus only on interventions with a true level of participation according stage model of participation by Wright et al., meaning that participants had at least to be involved in the decision-making process, and interventions that follow a digital approach at all stages of the participative elements. Specific aims are to describe (1) the participatory elements that were used in the interventions, (2) how the digital formats were used to conduct health promotion and prevention activities ensuring participatory approaches, and (3) benefits and limitations mentioned in relation to the digital format.

## Methods

We conducted a scoping review to identify existing literature on digital formats that enable participation in the field of health promotion and prevention in community settings. The aim of a scoping review is to map existing literature on a specific topic and to identify characteristics and concepts behind it (15). In contrast to a systematic review, the focus is not on the synthesis of results on a specific question, e.g., the effect of an intervention, but on providing an overview and a description of the field of research. As another goal is to identify the range of evidence on the topic, different study designs may be included and a formal assessment of the methodological quality of the included studies is usually not performed (16).

This scoping review was based on the framework of Arksey and O'Malley (17) and incorporated suggestions for improvement from Levac et al. (18), Daudt et al. (19) and the Joanna Briggs Institute (20). Additionally, the preferred reporting items of the PRISMA checklist for scoping reviews (PRISMA-ScR) (21) were taken into account.

The following steps were conducted:

Step 1: Identifying the research question
Step 2: Identifying relevant studies
Step 3: Study selection
Step 4: Charting data
Step 5: Collating, summarizing and reporting results

Arksey and O'Malley suggest an optional Step 6 where practitioners and consumers can be consulted (17). This step was not performed in this review.

A protocol was developed a priori by the author team, following the same guidelines as described in this scoping review. The final protocol was registered with the Open Science Framework on February 21, 2021 (https://osf.io/v9jfc/).

### Step 1: Identifying the Research Question

This scoping review was guided by the question: What kind of literature does already exist on digital formats that enable participation in the field of health promotion and prevention in community settings? Specific questions were: (1) Which participatory elements were used in the intervention? (2) How were digital formats used to conduct health promotion and prevention activities ensuring a participatory approach? (3) What benefits and limitations were mentioned related to the digital format?

We focused on the participation levels six to nine, according to the stage model of participation by Wright et al. (10). It defines true participation as participants being involved in the decision-making process or reaching a level of self-management. Furthermore, we were looking for an end-to-end digital approach that would be maintained throughout all or most sequences of the participation process, so that it would be feasible even in times of contact restrictions, as with the coronavirus pandemic.

### Step 2: Identifying Relevant Studies

Literature searches were conducted in the databases Medline, EMBASE, and PsycINFO. The search strategy for the database search included database-controlled vocabulary and additional keywords, using truncations to search by title and abstract. The search strategy was identical for all three databases, adapted to the respective subheadings of the database and contained the following terms: communit^*^ OR municipal^*^ OR citi^*^ OR city OR local OR neighborhood OR rural OR urban AND community participation OR participatory research OR participat^*^ OR empower^*^ OR involve^*^ OR engage^*^ OR partnership AND internet OR mobile applications OR social media OR smartphone OR mobile OR online OR digital OR photo^*^ OR video^*^ AND health promotion OR public health OR prevention OR health education.

Given the rapidly changing technological developments over the last decade, the search was limited to publications from 2010 to current studies (up to date when search was performed: 11/13/2020). Only studies in English were included.

Hand searches were additionally performed from the reference list of relevant reviews identified during the title and abstract screening to search for literature that may not have been captured by the databases used. After the search, duplicates were removed in the *EndNote* citation management software.

### Step 3: Study Selection

The third stage of Arksey and O'Malley's framework aims to identify the studies to be included in the scoping review. To maintain clear congruence between title, objectives, and research question, we based the inclusion and exclusion criteria on a PCC framework, consisting of the categories: population, concept, and context. “Study design” was added as an additional category (20).

We searched for members of a community, including geographically defined communities, communities connected through common interests or lifestyles or virtual communities. We focused on intervention or research concepts that used digital formats in all, or in the main sequences of the process for enabling participation or empowerment in the context of health promotion or prevention. Any empirical study designs except reviews or meta-analysis were included. The inclusion and exclusion criteria are shown in Table 1.

**Table 1:** Inclusion and exclusion criteria.

**Category**	**Inclusion criteria**	**Exclusion criteria**
Population	Members of a community, including geographically defined communities, communities connected through common interests or lifestyles or virtual communities	Institutional settings (e.g., health care settings, school settings, or occupational settings), clinical populations
Concept	Interventions or research methods that use digital formats in all, or in the main sequences of the participatory process Participation is defined as level 6–9 of the stage model of participation by Wright (10)	Interventions or research methods that - use analogous formats in all, or in the main sequences of the participatory process - only allow participation under level 6 of the stage model of participation by Wright, Unger and Block (10)
Context	All actions and programs relating to health promotion or health prevention	Actions and programs not related to health promotion or health prevention
Study design	Any empirical study design	- Non-empirical studies (e.g., commentaries, letters, editorials, recommendations, guidelines or overviews) - Reviews or meta-analyses

An iterative team approach was used for the screening process, which was conducted in two phases: first a title and abstract screening and second a full text screening with all included studies from the first phase. Prior to the initial title and abstract screening, an iterative process was used to screen the first 100 citations and discussed by the research team (MC, CJS, CS, SV) until inclusion and exclusion criteria were sufficiently specified. For the screening of records, one author (CS) screened 100% of the citations. The other authors divided 50% of the citations among each other, resulting in half of the records being double-screened independently. For the screening process, the web-based literature management program *Rayyan* was used. Conflicting reviewer decisions whether a study met the inclusion criteria were discussed and resolved in the team.

In the following full-text screening, a similar approach was used, resulting in 100% of the records being double-screened independently. Again, all conflicting classifications were discussed and resolved within the team. To ensure that no articles were missing, reference lists of relevant reviews were screened by the first author by title and abstract, and in full-text where appropriate.

The selection process is illustrated by a PRISMA flow chart displaying the results of the screening steps.

### Step 4: Charting Data

In the fourth stage of the scoping review framework, data was extracted from the included studies using an *Excel* spreadsheet. Following the recommendations from Levac et al. (18), the data extraction framework was developed collaboratively by the research team and iteratively updated. Four of the included studies were extracted independently by two authors and then discussed to test the coherence of the framework.

The extraction items where categorized into general information and specific information. The extracted data of the general information section were author, title, journal, year, country, health topic, study type, objectives, population (age, sex, characteristics, total number, setting), type and description of intervention, recruitment methods, data collection, sample size, analysis method, and findings.

The data extracted for the specific information section were type of digital elements and further detailed description of these elements, type of participatory activities and further description (such as level of participation) and benefits, limitations and conclusions related to the use of the digital formats.

### Step 5: Collating, Summarizing, and Reporting Results

The results were collated and summarized based on relevance to the research questions and reported in three tables. The first and second table focus on general information about each study, with the first table providing preliminary information and the second table presenting details about the methodology and results. The third table focuses on answering the three specific research questions. It contains information about the interventions related to the participatory aspect, the digital format, and the experiences made with it. The presented tabular results are accompanied by a narrative summary.

## Results

**Figure 1:**
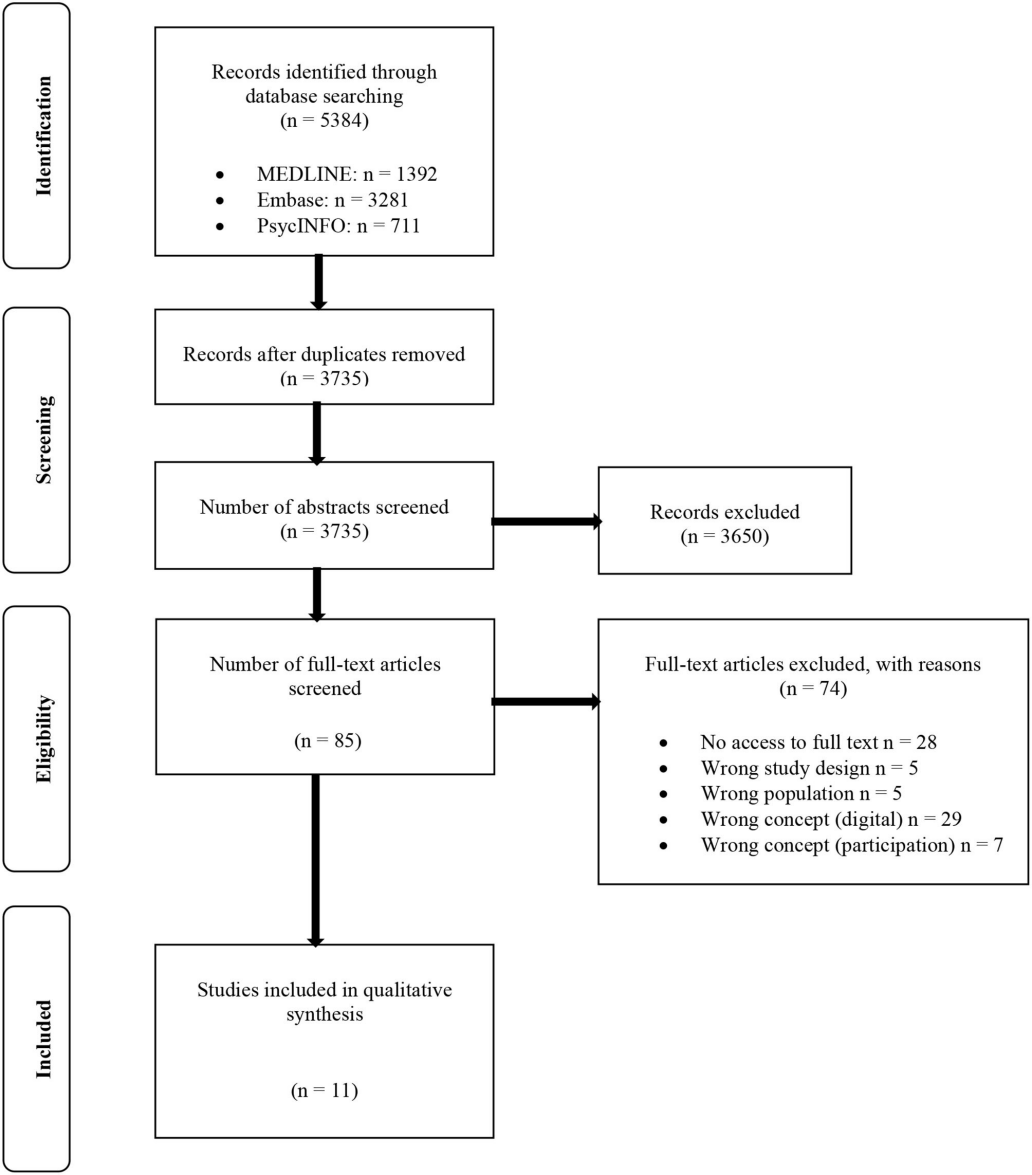
PRISMA flow chart according to Page et al. (33).

A total of 5,384 articles were found through database searching. After duplicates were removed in *Endnote*, 3,735 articles remained for title and abstract screening. The reference lists of eight relevant reviews (22–29) identified in the title and abstract screening were additionally screened, but no priorly unknown relevant study was found. From this initial screening phase, 85 articles were included in the full-text screening. Eleven studies were finally identified that met our inclusion criteria. The most common reasons for exclusion in the full-text screening were “no access to full text” (*n* = 28), mostly because only abstracts were available, and “wrong concept in relation to the digital format” (*n* = 29). Of the included 11 studies, three reported on the same intervention (30–32). The process of study selection is shown in the PRISMA flow chart (Figure 1) (33).

### Characteristics of Included Studies

Table 2 provides general information on the 11 included studies on publication year, country, study type, health topic addressed, and the study objectives.

**Table 2:** General information about included studies.

**First author**	**Year**	**Country**	**Study type**	**Health topic**	**Objectives**
Barry et al. (34)	2018	USA	Qualitative study, based on RCT	HIV prevention	To understand how resilience processes are shared among young Black GBMSM.
Bridges (35)	2016	Australia	Qualitative study	Breastfeeding support	To advance understanding of the experiences of mothers using closed Facebook groups for breastfeeding support.
Hildebrand et al. (36)	2013	Switzerland	Qualitative study	HIV prevention	To present a focused thematic analysis of a sub-section of the views expressed in the online and offline forums.
Iantaffi et al. (37)	2015	USA	Qualitative study	HIV prevention	To examine the acceptable level of sexual explicitness in HIV prevention advertisements.
Ripat and Colatruglio (38)	2016	Canada	Qualitative study	Wheeled mobility devices	To gain understanding of what people who use wheeled mobility devices identify as environmental barriers to community participation in cold weather climates.
Rothpletz-Puglia et al. (39)	2013	USA	Feasibility study	Health promotion	To offer opportunity and support for women at risk for or living with HIV to identify, create, and provide health promotion messaging within their informal personal networks.
Snider et al. (40)	2010	Canada	Feasibility study	Violence prevention	To engage youths, parents, and community workers in conceptualizing a hospital-based violence prevention intervention and to identify outcomes relevant to the community.
Ybarra et al. (41)	2014	USA	Qualitative study	HIV prevention	To examine self-reported behavioral and attitudinal changes among GBQ adolescent males who took part in online focus groups.
Young et al. (30)	2015	USA	Cluster randomized controlled trial	HIV prevention	To examine the efficacy of using the HOPE social media intervention to increase HIV testing among MSM in Peru.
Young et al. (31)	2014	USA	Randomized controlled trial	HIV prevention	To assess whether changes in network growth are associated with increased HIV prevention and testing behaviors.
Young et al. (32)	2013	USA	Cluster randomized controlled trial	HIV prevention	To determine whether social networking communities can increase HIV testing among African American and Latino MSM.

The included studies were published between 2010 and 2018. Seven studies were from the USA (30–32, 34, 37, 39, 41), noting that three studies were from the same research group, two were from Canada (38, 40), one from Australia (35), and one from Switzerland (36). Most studies (*n* = 6) followed a qualitative study design (34–38, 41), two were feasibility studies (39, 40), and the three studies from Young et al. were randomized controlled trials (30–32). Various public health topics were covered in the studies, such as breastfeeding (35), wheeled mobility devices (38), violence prevention (40), and health promotion in informal communities (39). The most common topic was HIV prevention, which was addressed in five of nine unique studies (30, 34, 36, 37, 41). The study objectives of the qualitative studies were all related to exploring the perspectives of the participants and therefore gaining a deeper understanding of the topic under research. The feasibility studies described the envisaged aims of their project and the RCT studies intended to measure the effectiveness of the intervention compared to a control group.

Table 3 presents general information of the 11 included studies regarding the target population and community setting, the data collection sources, the analysis methods, information on the participants of the sample and the main results.

**Table 3:** Methods and results of included studies.

**Author, year**	**Target population and setting**	**Data collection**	**Analysis method**	**Sample participants**	**Findings**
Barry et al. (34)	Age: 18–30 Sex: males Setting: Black, gay, bisexual MSM, recruited in southeastern US	Posts on the intervention website	Qualitative content analysis	Total number: 48 (RCT: 474) Average age: 24.3 years	Findings illustrate the width of roles that peer-level support played in fostering resilience. Self-acceptance and sex-positive norms were identified as new subthemes.
Bridges (35)	Age: all ages Sex: females Setting: breastfeeding mothers, members of closed Facebook groups attached to Australian Breastfeeding Association	3 online interviews, 3 online focus groups	Thematic analysis	Total number: 23 Administrators: 3 Group members: 20 Average age: not collected	Online breastfeeding support groups provide primarily support from a trusted community. Social networking sites are further described as immediate, complementary to existing support services, and a source of information for users.
Hildebrand et al. (36)	Age: 15–29 Sex: all genders Setting: young people of 79 countries, connected to the UNAIDS network	Data from a secondary analysis of the themes and texts that emerged during the UNAIDS discussions	Focused thematic analysis	Total number: 5,102 Online forums: 3,497 Offline forums: 1,605 Average age: not collected	Youth identified the need to change the way sex and relationships are dealt with through comprehensive sexuality education, overcoming social and cultural taboos, and changing how sex is talked about.
Iantaffi et al. (37)	Age: 18 or older Sex: males Setting: MSM regularly viewing sexually explicit media, living in the US	13 online focus groups	Content analysis	Total number: 79 Average age: 18–44 years (most participants were white, HIV-negative men)	The acceptable level of sexual explicitness in HIV prevention campaigns depends on factors of audience, location, and community representation.
Ripat and Colatruglio (38)	Age: adults Sex: all genders Setting: users of wheeled mobility devices (WMD), residents of Manitoba, Canada	1 online asynchronous focus group	Content analysis	Total number: 8 (7 wheelchair users, 1 scooter & walker user)	“Study confirms that elements of the environment, including the natural environment, supports, services, policies, and WMDs can alternatively serve as a barrier or facilitator to community participation.” (p. 102)
Rothpletz-Puglia et al. (39)	Age: adults Sex: females Setting: women at risk for or living with HIV, living in New Jersey	Self-reported activity logs, participant questionnaires, and community-recipient evaluations	Mixed methods approach	Total number: 57 in-person group: 38 online group: 19	Women in both groups successfully provided health promotion to 5,861 people in their social networks. This demonstrates the feasibility of building social networks for disseminating health information and reducing health disparities in communities.
Snider et al. (40)	Age: youth and adults Sex: all genders Setting: youths, parents, and community youth workers, recruited in Toronto	Data was collected through 'The Concept System' software	Cluster analysis	Total number: 278 Brainstorming: 48 Sorting: 103 Rating: 102 Interpretation: 25 Average age (if youth): 12–24	It is feasible to use information generated by youth to develop successful and meaningful interventions to prevent youth violence.
Ybarra et al. (41)	Age: 14–18 Sex: males Setting: gay, bisexual, and queer males, recruited nationally (USA)	4 questions subsequent to online focus groups	Thematic analysis	Total number: 75 Group with no sexual experience: 36 Group with sexual experience: 39	The majority reported that their participation positively changed their views and behavioral intentions about their sexuality. Sexually inexperienced youth most commonly reported positive effects of feeling less isolated.
Young et al. (30)	Age: 18 or older Sex: males Setting: Participants and peer leaders are MSM with a Facebook account, living in Lima (Peru). Participants should be HIV negative or serostatus unknown.	Baseline survey before the intervention	Multivariate adjusted logistic regression	Total number: 498 Intervention group: 252 Control group: 246 Peer leaders: 34 Average age: 28.9 years	Participants in the HIV intervention groups were more likely to request an HIV test than were those in the control groups. Peer-mentored social media communities seemed to be an effective method to increase HIV testing among high-risk populations in Peru
Young et al. (31)	Age: 18 or older Sex: males Setting: Participants and peer leaders are MSM with a Facebook account, living in Los Angeles. Peer leaders should be African American or Latino.	Baseline and follow-up survey, participants' FB friend lists	Network visualizations, regression analyses	Total number: 105 Peer leaders: 16 Average age: 31.5 years (90% were Latino or African American)	Among the intervention group, a positive trending relationship between increased network ties and likelihood of HIV testing, follow-up for test results, and participation in online community discussions was found.
Young et al. (32)	Age: 18 or older Sex: males Setting: Participants and peer leaders are MSM with a Facebook account, living in Los Angeles. Peer leaders should be African American or Latino.	Baseline and follow-up survey	Chi-square tests, *t*-tests, rates of home-based HIV testing	Total number: 105 Peer leaders: 16 Average age: 31.5 years (90% were Latino or African American)	“Social networking communities are acceptable and effective tools to increase home-based HIV testing among at-risk populations.” (p. 2)

The target population were adolescents or young adults in three studies (34, 36, 41), adults aged 18 years or older in six studies (30–32, 37–39) and adolescents and adults in one study (40). One study did not specify its target population (35). All studies focused on community settings defined by common identity. Four of the nine unique studies additionally searched for a geographic community setting by looking for residents of a specific city (30, 38–40). The remaining studies had no specific geographic setting or only a widely defined one. One study targeted virtual communities (35).

Of the six qualitative studies, four collected data using online focus groups (35, 37, 38, 41) and two *via* written discussions from online forums (34, 36). One study conducted in-depth interviews in addition to focus groups (35). Three of these studies performed content analyses (34, 37, 38) and three conducted thematic analyses (35, 36, 41). Snider et al. (40) used a concept mapping software for collecting the data and performed a cluster analysis. Rothpletz-Puglia et al. (39) used various sources for data collection, such as self-reported activity logs and participant questionnaires, and applied a mixed methods approach. Young's studies also used multiple data sources, with baseline and follow-up surveys in each study, and conducted a mix of quantitative analysis methods (30–32).

The number of participants in the focus group studies varied between eight and 79 participants. The crowdsourcing project involved a total of 5,102 participants (36) and Snider's (40) concept mapping project a total of 278. Two studies by Young et al. reported the same sample (31, 32). These had a total number of 105 participants. The other study sample had a total number of 498 participants (30). All studies that conducted content or thematic analysis reported a deeper understanding regarding their study objectives. The two studies testing feasibility were able to confirm and reported a successful implementation and acceptability of their intervention (39, 40). The studies by Young et al. that tested a peer-mentoring intervention were able to confirm its effectiveness compared to a control group.

### Types of Participation

Table 4 maps specific information of the interventions including a brief description of the intervention (if applicable), a more detailed description of the participatory elements, information about the type of digital format and how it was used, and a collection of strengths and weaknesses mentioned in relation to the digital format. The three studies by Young et al. (30–32) are combined in this table as they report the same intervention.

**Table 4:** Characteristics of interventions.

**Author, year**	**Description of intervention**	**Type of community participation**	**Description of participatory activities**	**Type of digital element**	**Description of digital elements**	**Benefits related to the digital format**	**Limitations related to the digital format**
Barry et al. (34)	HealthMpowerment is an online intervention that aimed to reduce condomless anal intercourse and foster community among young Black GBMSM. In online forums the intervention group could react to pre-populated or staff-generated conversations (control group got an information-only website).	Peer support *via* online forums	Online forums created spaces for participants to connect and support each other, which fostered resilience processes. Topics for the pre-populated or staff-generated conversations were identified through formative research with young Black GBMSM.	Website (mobile phone optimized), which provided two online forums (*The Forum* and *Getting Real*) for participant interaction	“*The Forum* was a space where participants could read and contribute to existing conversation threads or start new threads. *Getting Real* was designed to be a creative space where participants could post and respond to topics with videos, poems, reflections, audio, and pictures.” (p. 4)	- Anonymity (facilitated more candid questions and discussions than might have been provided in-person) - Flexibility to choose how and what to contribute - Close to real life interactions because interpersonal interactions increasingly take place online, particularly among stigmatized groups	“It can be difficult to interpret the tone and intention of online data.” (p. 14)
Bridges (35)	/	Participatory research *via* online in-depth interviews and online focus groups	In the interviews and focus groups, participants were asked about their experiences administering or participating in closed Facebook groups for breastfeeding support.	Social media platform *Facebook* used for qualitative interviews and focus groups	Interviews were conducted *via* the “Messenger” function. Questions were typed in and answered asynchronously or synchronously by the participants. The focus groups were conducted *via* the “Event” function. All questions were created as wall posts and answered asynchronously by participants *via* the “comment” function.	- Asynchronous conduction: participants could answer any time they wanted in the interviews and focus groups - Easy accessibility *via* Facebook: participation possible from any location(both points helpful as participants were mothers of young children)	/
Hildebrand et al. (36)	CrowdOutAIDS is a participatory online policy project using a crowdsourcing process. It consists of 4 steps to enable young people to both formulate the problems as well as generate solutions in the AIDS response, resulting in a strategy document for the UNAIDS Secretariat.	Crowdsourcing process to enable policy participation	1) A community of interested young people were connected 2) Young people could share their experiences, ideas, and information 3) Participants were enabled to find solutions *via* voting 4) Final strategy document was co-authored in public online sessions, and a drafting committee of young people worked with the UNAIDS Secretariat to implement the strategy	1) Social media and online platform2) Social networking platforms: *Facebook, RenRen* and *Vkontakte*3) Customized osqa.net application4) Customized GoogleDocs application	1) Initial buzz *via* social media and implementation *via* the online platform CrowdOutAIDS.org 2) 9 regional online forums, conducted *via Facebook, RenRen*, and *Vkontakte* 3) Customized osqa.net application for voting 4) Customized GoogleDocs application (no further description provided)	- The digital crowdsourcing processes facilitates the integration of grassroots perspectives from across the globe, engagement, and participation. - Enabled participation beyond youth networks and organizations to involve “ordinary” young people - “Despite the digital divide, online tools can effectively be used to mobilize for offline action.” (p. 67)	- Digital divide (counteracted in the 2nd step through offline forums) - Selection bias: Online participants had a higher socioeconomic background
Iantaffi et al. (37)	/	Participatory research *via* online focus groups	Participants were asked to give their opinions on HIV-prevention poster advertisements and one video advertisement	Adobe Connect used for online synchronous focus groups, bulletin board for asynchronous follow-up questions	The synchronous focus groups were conducted *via* the “chat” function, without the use of audio or video. After conclusion, participants were invited to respond asynchronously to follow-up questions and comments posted on a message board.	Higher confidentiality, by not using audio or video functions.	Data is limited to written text and does not give access to audio or visual participants' reactions.
Ripat and Colatruglio (38)	/	Participatory research *via* online focus group	“The researchers were seeking a nuanced understanding of WMD users' experiences regarding community participation and winter barriers, the strategies they employ to overcome those barriers, and the recommendations they had for improving winter community participation.” (p. 97)	WordPress.com for hosting asynchronous online focus group	Participants had 1 week to respond asynchronously to daily questions posed by a moderator.	“Time for more in-depth and reflective responses from participants, greater participant anonymity, increased convenience in terms of participating from any location at any time, and automatic capture of discussion data” (p. 97)	/
Rothpletz-Puglia et al. (39)	Shout-out Health is a community-driven health promotion approach aiming to empower high-risk community members to develop and provide health promotion messaging delivered in their informal social networks.	Empowerment intervention for providing health promotion in informal social networks	Within in-person or online groups participants identified health problems and developed ways to promote health information in their informal social networks over a 5–6 week period. Therefor a 5-step intervention process was conducted.	Asynchronous online group (no information about the program)	Over a 3-month period, women in the online group worked asynchronously except for 2 conference calls. For the participants' convenience, they could choose the meeting format but could not move back and forth between the 2 approaches. (no further description provided)	- Convenience for participants to choose between online and in-person group - No significant differences in the productivity by group meeting format (p. 3)	/
Snider et al. (40)	/	Community-based participatory research using concept mapping	Opinions and ideas from community members about how hospitals could help youth avoiding violence in the future were collected, sorted, and rated online. In a face-to-face meeting, participants discussed the assessment results and drafted an intervention concept.	“The Concept System” software for conducting the online concept mapping processes	1) Brainstorming: participants were asked to enter statements in response to a prompt (8 weeks) 2) Sorting: online participant sorted the brainstormed statements into piles (6 weeks) 3) Rating: participants rated each statement in terms of importance (6 weeks)	- Anonymity (given the sensitivity of the subject) - Flexibility: “Participants could partake in any or all steps of the concept mapping process.” (p. 878)	- Due to anonymity, determination of how many participants took part in all four steps not possible - “A limitation of the software used in this study is a restriction on the number of demographic questions that can be asked.” (p. 883)
Ybarra et al. (41)	/	Participatory research *via* online focus groups	Participants took part in two rounds of asynchronous focus groups in which they discussed their ideas, thoughts, and concerns about an HIV prevention program. Afterward, they were asked 4 questions how their participation in the focus groups influenced or changed their views or behaviors.	Bulletin board used for asynchronous focus groups and the following questions	“Each day, questions were posted on the bulletin board in the morning and then again, in the afternoon. Participants were instructed to visit the board at least twice a day to respond to questions, reply to moderator probes, and interact in discussions with other group members.” (p. 3)	- Safe and anonymous environment, in which participants could talk more freely about their sexuality - Online focus groups represent a low-cost, scalable intervention	/
Young et al. (30–32)	The HOPE Social Media Intervention tested whether MSM in Facebook groups with peer-mentored HIV prevention and behavior change information would be more likely to test for HIV than those in groups without a peer-leader.	Peer-mentoring and peer support	MSM who were described as well-respected among the MSM community were trained as peer-leaders. They were advised to communicate with their assigned participants on Facebook in addition to general “friendly” conversation about HIV prevention and testing.	Social media platform *Facebook* used for conducting online intervention	Private Facebook groups consisted of 30 participants and 4–6 peer leaders who communicated by sending messages, chats, and wall posts. Participants had no obligations to respond or to stay in the Facebook group.	- Low costs HIV solution - Broader reach than traditional public health interventions - Reduce travel and time costs - Easy implementation - Growing international popularity of social media - Easier data collection than in field settings	- Duplicate respondents found during recruiting (non-unique usernames)

We found five studies that conducted qualitative participatory research. As reported above, four of them used online focus groups as research method (35, 37, 38, 41). One study additionally conducted in-depth interviews (35). In the focus groups and interviews, the researchers sought a nuanced understanding of the participants' views and perspectives on a particular issue affecting their lives. Participants were asked about their experiences, opinions, ideas, thoughts and recommendations. One study used concept mapping as a community-based participatory research method (40). Through this process, community members were enabled to gather their opinions and ideas about how hospitals could help youth avoid violence in the future. After that participants could sort and rate these ideas through an online process. In a face-to-face meeting, these results were developed into an intervention concept.

One intervention enabled peer support using online forums where participants could connect and support each other by starting conversation threads or responding to staff-generated threads (34). Another intervention that also used a peer support approach, trained peers of a community to become peer mentors who provided HIV prevention by communicating with the participants through *Facebook* groups (30–32).

We found one study that implemented an empowerment project providing opportunity and support for women at risk or living with HIV to identify, create and deliver health-promoting messages in their informal personal networks (39). To facilitate this project, a partnership was established between an academic and community agency.

One study used a crowdsourcing method to allow young people to participate in policy decision-making by both formulating the problems and generating solutions for the AIDS response (36). Similar to the concept mapping process, participants were able to share ideas and vote on what should be included in a strategy paper for the UNAIDS Secretariat, which was created through a public co-authoring process.

### Utilization of Digital Formats

Online focus groups were mostly conducted in an asynchronous manner, meaning that participants did not have to respond immediately. Bridges (35) used the social network platform *Facebook* for both focus groups and interviews. Focus group questions were posted on a wall using the “event” function, interview questions were typed in *via* the “messenger” function. Iantaffi et al. (37) used *Adobe Connect* for conducting synchronous focus groups *via* the “chat” function, without the audio or video function, and an asynchronous bulletin board for follow-up questions. The online focus group of Ripat and Colatruglio (38) was also conducted in an asynchronous manner by commenting on questions from a moderator hosted on *WordPress.com*. Ybarra et al. (41) used a bulletin board to conduct the focus groups and the subsequent behavior change questions. Participants were able to respond to questions, reply to moderator probes and interact in discussions with other group members.

Barry (34) used a website that was later optimized by a mobile phone application, which offered two online forums. In both forums, participants had the opportunity to read and contribute to existing conversation threads or start new threads and to post and respond to topics with videos, poems, reflections, audio, and pictures.

The HOPE Social Media Intervention was conducted *via* the social networking platform *Facebook* by creating closed groups in which the peer mentors could communicate with the participants by sending messages, chats, and wall posts (30–32).

Snider et al. (40) used a special program called *The Concept System* for the concept mapping process, in which participants could brainstorm ideas online, sort them, and rate them over a period of several weeks.

The CrowdOutAIDS program used various online applications for its four-step crowdsourcing process. Participants were recruited *via* social media and directed to the CrowdOutAIDS.org website. Online forums were then conducted through the social networks *Facebook, RenRen* and *Vkontakte*, and additional community forums were organized through online participant volunteers. Statements abstracted from the forums were voted for *via* a customized *osqa.net* application. In subsequent public online sessions, the final strategy document was created in a co-authoring process *via* a customized *GoogleDocs* application. A more detailed description of the applications was not provided.

The Empowerment project by Rothpletz-Puglia et al. (39) offered a choice between online and in-person groups. The online group worked asynchronously except for two conference calls. More information about the digital format and its usage was not provided.

### Benefits and Limitations Associated With Digital Approach

One of the strengths of online forums is that they offered anonymity, which facilitated discussion of more candid questions (40). It was also noted by Barry et al. (34) that communication *via* online platforms came close to their real-life interactions and was therefore familiar. In addition, the flexibility for participants to decide how and what to contribute was mentioned as a strength. As a limitation, the difficulty of interpreting the tone and intention of online data was noted.

Similar points were mentioned in the study from Iantaffi et al. (37). The anonymity of online focus groups offered higher confidentiality by not using audio or video functions, but the lack of access to audio or visual participants' reactions made the interpretation of the data more difficult.

Snider et al. (40) concluded that anonymity is a strength of the online concept mapping process, which was particularly valuable with regard to the topic of experienced violence. Also, the flexibility of allowing participants to decide for themselves whether they wanted to participate in any or all steps was cited as a strength. The disadvantage of this procedure, however, was that it was not possible to evaluate how many participants took part in all steps of the process.

Ybarra et al. (41) also mentioned that the online format created a safe and anonymous environment that allowed participants to talk more freely about their sexuality. Moreover, it is added that online focus groups can represent a scalable low-cost intervention.

The social media intervention The HOPE was also identified as a low-cost solution for HIV prevention that enabled a broader reach than traditional public health interventions, was easy to implement, reduced travel and time costs and facilitated data collection (30–32). A disadvantage of social media interventions was cited as finding duplicate respondents during recruitment through non-unique usernames.

Hildebrand et al. (36) highlighted the broader reach of digital methods as a strength, enabling the integration of grassroots perspectives from across the globe and the involvement of young people who would not have normally been involved through traditional participatory processes. As a limitation of digital formats, Hildebrand mentioned the digital divide and the associated selection bias, as it was assumed that online participants have a higher socioeconomic background.

Conducting the asynchronous focus groups and interviews *via Facebook* was beneficial for easy accessibility and flexibility for participants to decide for themselves the time and place they want to respond (35). Especially for young mothers this format was considered suitable.

Ripat and Colatruglio (38) mentioned the increased convenience in terms of participating from any location at any time given by online focus groups, in addition to anonymity, the automatic data collection, and the fact that participants have time to give more reflective responses.

Rothpletz-Puglia et al. (39) compared the productivity between online groups and in-person groups and found no significant differences. They described the possibility to choose between both formats as a convenience for participants.

## Discussion

With this scoping review we mapped the existing literature on digital formats that enable participation in the field of health promotion and prevention in community settings. Furthermore, we gained a more comprehensive understanding of core concepts in this research area in terms of the enabled types of participation, the ways of utilizing the digital formats and the benefits and limitations linked to it. We identified nine unique out of 11 included studies relevant to the research question. Although these are a relatively small number of studies, they seem to reflect to some extent the diversity of methods and topics as well as certain trends in the literature.

### Characteristics of Included Studies

The majority of included studies dealt with vulnerable populations, mostly targeting communities at risk for or living with HIV. The huge amount of studies focusing on community-based HIV prevention programs and activities was also noticeable in the title and abstract screening. This may indicate that digital interventions are often used to reach stigmatized or vulnerable groups who commonly face challenges in seeking support. The easier accessibility and anonymity are particularly beneficial for reaching those populations (42).

Only youths and younger adults between 12 and 44 years were targeted or included in the interventions of our selected studies. This may reflect the fact that this age group can be reached better and more easily using digital media than older adults. As interpersonal interactions of younger adults increasingly take place online, digital interventions more closely resemble their real-life interactions than they do for older people, who generally have less exposure to digital media and lower levels of e-health literacy (34, 43).

### Types of Participation

We found three studies that used community participation methods, like crowdsourcing and concept mapping, to enable large groups of people to actively define relevant issues for their community themselves and help shape solutions. We also found participatory research studies that mostly used a focus group approach. Through this method, participants were able to express their views and perspectives on a specific issue affecting their lives. The deeper insights into participants' experiences, opinions, thoughts, and recommendations not only benefited the researchers in generating more detailed data. Through participation in focus groups, community members reported benefits including new insights and a broader perspective on issues shared within the group, in addition to a sense of inclusion and community building (44). This was most evident in the study by Ybarra et al. (41), which found that online focus groups discussions should be further explored as low-cost prevention programs.

### Utilization of Digital Formats

The digital technologies used varied widely, from social media platforms to customized web providers and programs. Digital technologies were used in most cases to establish direct communication between researchers and participants or to establish communication between participants. In the crowdsourcing, and concept mapping interventions, digital formats were used for mass community engagement that enabled democratic processes such as voting, brainstorming, sorting and rating. These findings demonstrate that digital technologies can, on the one hand, facilitate communication between community members and thus foster social networks. On the other hand, they can be used to create open environments to involve large groups of individuals for community participation processes (45). Crowdsourcing, in particular, is a method of mass collaboration that increases in the field of public health (27).

### Benefits and Limitations Associated With Digital Approach

Three studies found that the interaction with like-minded people in online spaces increased feelings of support and self-acceptance among participants and reduced feelings of isolation (34, 35, 41). It was stated that the anonymity facilitated by the digital formats was beneficial to these interactions, as well as the easy and immediate access to support and flexibility to decide how much and when to participate. This is congruent with the reasons why digital peer support formats are increasingly offered in the field of mental health identified by a systematic review (46). Most of our identified qualitative participatory research studies used online asynchronous focus groups. For this method it is essential to create a “safe space” where participants have a feeling of confidentiality to express their opinions and experiences freely. The frequent choice for asynchronous formats could be an indication that this necessary environment can be created particularly well by this approach, by allowing a higher degree of anonymity and flexibility for participants to determine when and where to respond.

None of the included studies mentioned ethical aspects and data protection issues as a limitation or barrier of digital formats. This may be due to the fact that the majority of studies were from Anglo-American countries. This should be taken into account when transferring the study concepts to countries with high data protection requirements, such as Germany. Only five studies reported limitations related to the digital format, including difficult interpretation of written data. Previous research has already indicated that sufficient written literacy must be a criterion when conducting interventions limited to written discussions (47). This carries the risk of excluding certain groups of people, which is why suitability for the target population needs to be considered. In the title and abstract screening, we also found many studies on photovoice that were often not included because the subsequent focus group discussions were mostly conducted in a non-digital format. Since photovoice is less about examining participants' views on something and more about creating an impact, face-to-face formats may be better suited for this purpose (48).

Existing studies on digital interventions in various community settings mention several benefits compared to traditional formats. A review by Gilbey et al. (42) outlined in regard of the LGBTQ (lesbian, gay, bisexual, transgender, queer) community the anonymity aspect of digital interventions which facilitates access to support by minimizing stigma. Wadham et al. (49) found that specific communities like adolescents and young adults can be easier reached with new digital media interventions, because they use digital ways for information sharing on a regular basis. A review by Fortuna et al. (46) mentioned that digital peer support is increasingly delivered through social media networks, smartphone apps, and technologies that enable synchronous and asynchronous communication. This allows for a wider reach of peer support services. Digital interventions also become increasingly popular due to easy implementation, cost-effectiveness, and remote accessibility (50). On the other side, higher drop-out rates and a lack of evidence on the effectiveness of digital interventions have been reported (42, 51). Additionally, according to Hall et al. (52), aspects of “poor technology skills, interfaces that are not user-friendly, concerns around data security, and a lack of support from healthcare professionals” should be considered when implementing digital interventions.

The COVID-19 pandemic resulted in the implementation of certain non-pharmaceutical measures, such as cancelling public events, restricting social gatherings to a minimum and closing schools and workplaces (53). Social distancing demonstrated the need and potential for using digital solutions with a consistent digital approach to engage people in health promotion and prevention activities.

However, there are some open questions with regard to the implementation of digital formats in community-based health promotion interventions which should be focused on in future research activities. It should be considered carefully whether digital formats could replace traditional formats for health promotion and prevention activities especially in vulnerable populations. From our perspective, one of the most urgent unanswered questions is whether the implementation of digital health promotion interventions leads to a further increase of a selection bias or whether such interventions counteract this bias and are used and accepted by vulnerable groups and settings in which traditional formats fail.

### Strengths and Limitations

This review contains some methodological limitations. First, we may have missed some relevant studies related to the research questions for a few reasons. Our search was restricted to three databases without including grey literature. We searched for publications in English only and therefore might have missed studies published in other languages from geographic areas where digital public health technologies are known to be widely used (e.g., Korea, Japan). There were also a high number of studies that had to be excluded because the full texts were not available. In addition, we conducted the database search in November 2020, which may have been too early to find relevant studies related to the COVID-19 pandemic. Second, we did not perform a quality assessment of the included studies and did not focus on ethical and data protection aspects of using digital formats. However, this was not considered relevant to the objective of this study, which was to collect existing literature on this topic and examine it for key concepts. Considering the relatively low number of included studies compared to the number of papers found by the literature search, this might be explained by the clearly defined and specific inclusion criteria, as we were looking for interventions with a high level of participation for communities and a high proportion of digital implementation in the intervention. This focus was also reflected in the search strategy, where we searched specifically for community, participation and their synonymous terms. These criteria were needed for narrowing the amount of publications found at the initial search. However, this may have led to the omission of publications that might have been of interest for our work. Despite these limitations, we do consider this clear focus to be a strength and a quality criterion of this paper.

Other strengths of this review were the sound methodology based on recommended frameworks of Arksey and O'Malley and the PRISMA checklist for preferred reporting items.

## Conclusion

This scoping review only found a few studies following a consequent digital format to enable a high level of community participation in health promotion and prevention, indicating an existing gap in research on this topic. Digital formats were found to be suitable for purposes where anonymity is helpful. In the included studies, this was apparent in qualitative participatory research studies, particularly in online focus groups that required participants to talk about sensitive subjects. Furthermore, the aspects of anonymity and easy accessibility appeared to be beneficial in supporting vulnerable and stigmatized communities, such as through peer exchanges and peer support programs. Further research should be conducted on the purposes for which digital formats can be more effective than traditional formats in enabling participation, in order to make more targeted use of the potential of digital technologies and social media. Disadvantages of digital formats, such as possible selection bias due to the digital divide and difficulties in interpreting written-only data, have to be weighed against the benefits. The consequences of social distancing due to the COVID-19 pandemic and the unpredictability of similar exceptional circumstances in the future stress the need to further develop and implement digital formats in health promotion and prevention activities in community settings.

## Author Contributions

CS conducted the database search. During the screening process, CS screened all records in the title/abstract and in the full-text screening stage. MC, SV, and CJ-S divided half of the records among each other during the title/abstract screening. In the full-text screening phase, MC and SV screened half of the records each. CS, SV, and MC were involved in the data extraction process. CS drafted the first version of this manuscript and developed it further based on feedback from SV and MC. CS, SV, CJ-S, and MC all read, reviewed, and approved of the final version of the manuscript. All authors contributed to the conception of the study and the development of the search strategy.

## Conflict of Interest

The authors declare that the research was conducted in the absence of any commercial or financial relationships that could be construed as a potential conflict of interest.

## Publisher's Note

All claims expressed in this article are solely those of the authors and do not necessarily represent those of their affiliated organizations, or those of the publisher, the editors and the reviewers. Any product that may be evaluated in this article, or claim that may be made by its manufacturer, is not guaranteed or endorsed by the publisher.

## Abbreviations

COVID-19: Coronavirus disease 19
FB: Facebook
GBMSM, Gay, bisexual: and other men who have sex with men
GBQ, Gay, bisexual: and queer
HIV: Human immunodeficiency virus
LGBTQ, Lesbian, gay, bisexual, transgender: queer
MSM: Men who have sex with men
WMD: Wheeled mobility devices.
